# n-Butanol production in *S. cerevisiae*: co-ordinate use of endogenous and exogenous pathways

**DOI:** 10.1007/s00253-018-9305-x

**Published:** 2018-09-01

**Authors:** R. Swidah, O. Ogunlabi, C. M. Grant, M. P. Ashe

**Affiliations:** Division of Molecular and Cellular Function, School of Biological Sciences, Faculty of Biology, Medicine and Health, Manchester Academic Health Science Centre, The University of Manchester, Michael Smith Building, Oxford Rd., M13 9PT Manchester, UK

**Keywords:** *S. cerevisiae*, n-butanol, Endogenous pathway, Exogenous pathway, Metabolic engineering

## Abstract

n-Butanol represents a key commodity chemical and holds significant potential as a biofuel. It can be produced naturally by *Clostridia* species via the ABE pathway. However, butanol production via such systems can be associated with significant drawbacks. Therefore, substantial efforts have been made toward engineering a suitable industrial host for butanol production. For instance, we previously generated a metabolically engineered *Saccharomyces cerevisiae* strain that produces ~300 mg/L butanol from combined endogenous and exogenous pathways. In this current study, the endogenous and exogenous pathways of butanol production were further characterised, and their relative contribution to the overall butanol titre was assessed. Deletion of any single component of the exogenous ABE pathway was sufficient to significantly reduce butanol production. Further evidence for a major contribution from the ABE pathway came with the discovery that specific yeast deletion mutants only affected butanol production from this pathway and had a significant impact on butanol levels. In previous studies, the threonine-based ketoacid (TBK) pathway has been proposed to explain endogenous butanol synthesis in *ADH1* mutants. However, we find that key mutants in this pathway have little impact on endogenous butanol production; hence, this pathway does not explain endogenous butanol production in our strains. Instead, endogenous butanol production appears to rely on glycine metabolism via an α-ketovalerate intermediate. Indeed, yeast cells can utilise α-ketovalerate as a supplement to generate high butanol titres (> 2 g/L). The future characterisation and optimisation of the enzymatic activities required for this pathway provides an exciting area in the generation of robust butanol production strategies.

## Introduction

n-Butanol (butanol) represents a key intermediate in the chemical industry with uses in the production of paints, adhesives, cosmetics, solvents and artificial flavouring. It also has wide potential as an alternative fuel to gasoline (Ndaba et al. 2015; Peralta-Yahya et al. 2012). For instance, in comparison with bioethanol, n-butanol is less hygroscopic, it is less corrosive, it has a higher energy content and it can be used at any ratio with gasoline, including directly as a fuel without engine or infrastructure modification (Cascone 2008).

**Fig. 1:**
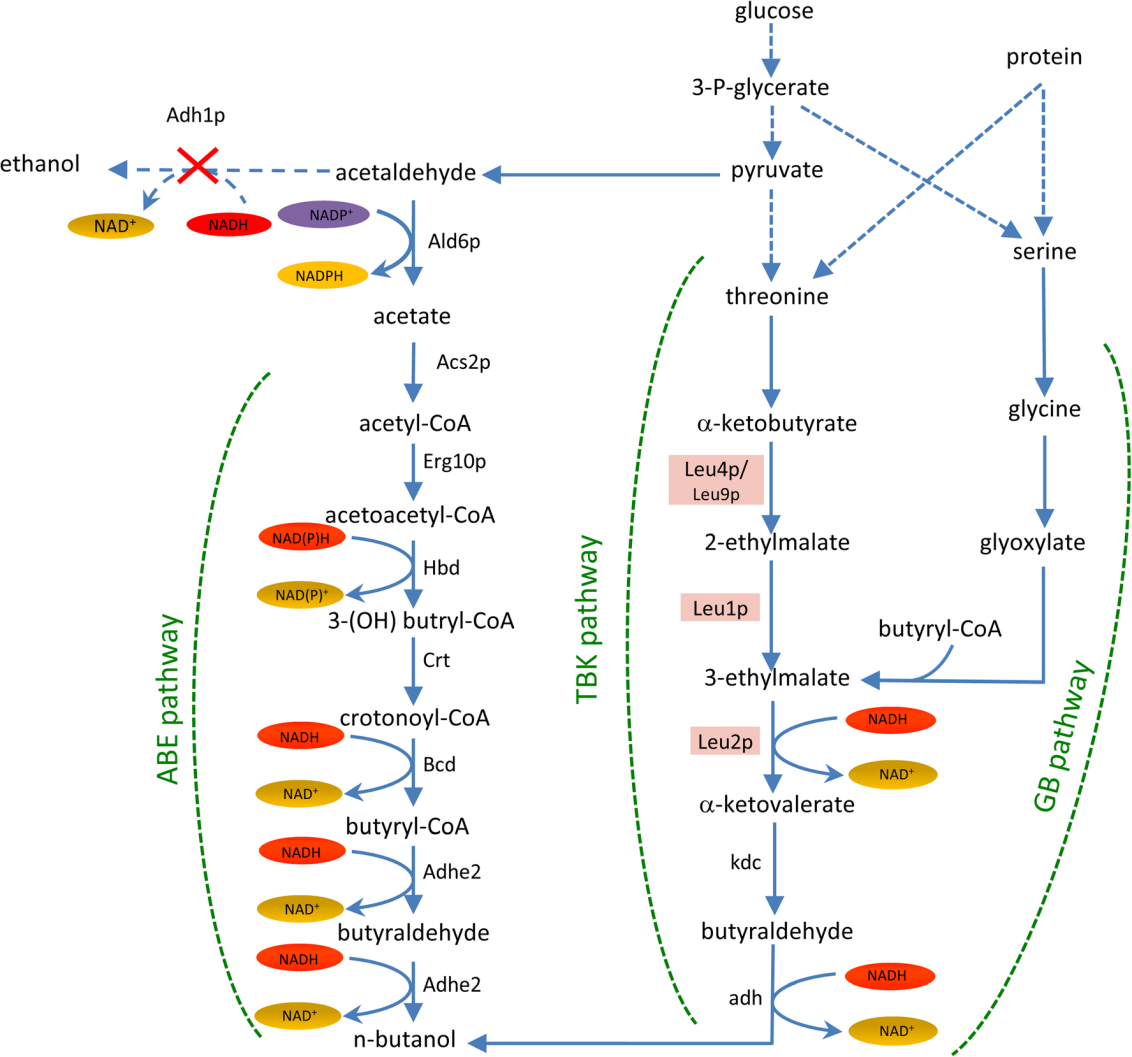
Endogenous and exogenous metabolic pathways for butanol production in *S. cerevisiae.* A schematic diagram of the exogenous acetone-butanol-ethanol (ABE) pathway enzymes, and the endogenous threonine-based ketoacid (TBK) and glycine-butanol (GB) pathways. Gene cassettes expressing components of the ABE fermentation pathway and upstream enzymes that have been inserted into the yeast genome are depicted: Hbd (3-hydroxybutyryl-CoA dehydrogenase), Crt (3-hydroxybutyryl-CoA dehydratase), Bcd (crotonyl-CoA reductase) and Adhe2 (alcohol dehydrogenase) from *Clostridium beijerinckii*, and Erg10p (thiolase), Ald6p (aldehyde dehydrogenase) and Acs2p (acetyl-CoA synthetase) from *S. cerevisiae*. The deletion of the yeast *ADH1* gene is also depicted as a red cross, and relevant components of the endogenous pathways are highlighted, Leu1p (Isopropylmalate isomerase), Leu2p (β-isopropylmalate dehydrogenase) and Leu4p/Leu9p (α-isopropylmalate synthase)

Traditionally, n-butanol has been produced from various *Clostridia* species via acetone–butanol-ethanol (ABE) fermentation (Lee et al. 2008). In the acidogenic exponential growth phase, acetate and butyrate are produced; then cells arrest growth and convert these acids to ethanol, butanol and acetone during a solventogenic phase (Patakova et al. 2013). The precise pathway for butanol production involves the condensation of two acetyl-CoA molecules to acetoacetyl-CoA followed by sequential reduction to n-butanol (Fig. 1). However, bacterial production can be associated with a range of difficulties, including butanol toxicity, bacteriophage contamination, the complexity of the two phase fermentation (acidogenesis and solventogenesis), sporulation during solventogenesis, high levels of by-products and very low titre at scale (Zheng et al. 2009; Hong and Nielsen 2012; Xue et al. 2013; Huffer et al. 2012). Therefore, a number of studies have focussed on producing butanol from alternative hosts*,* which may overcome some of the problems associated with the use of *Clostridia* species.

A body of work has demonstrated that various strategies can be used to engineer yeast such that they produce n-butanol. The introduction of the Clostridial ABE pathway into yeast results in a low titre of butanol (Steen et al. 2008; Swidah et al. 2015), but additional modifications can improve this titre. For instance, levels of butanol can be increased using strategies to increase the level of cytosolic acetyl-CoA. For instance, overexpression of the machinery involved in converting ethanol to acetyl-CoA (the *ALD6*, *ACS2* and *ADH2* genes), or deletion of the glyoxylate cycle components that normally deplete cytosolic acetyl-CoA (*CIT2* and *MLS1*) significantly improve butanol production (Krivoruchko et al. 2013; Swidah et al. 2015). Another strategy to improve butanol titre involves the restriction of competing pathways and restoration of redox balance caused by the introduction of the ABE pathway. Hence, deletion of the alcohol and glycerol dehydrogenase genes involved in ethanol and glycerol biosynthesis respectively has been shown to improve butanol titre (Lian et al. 2014; Schadeweg and Boles 2016; Swidah et al. 2015).

While studying butanol production from exogenous Clostridial pathways in yeast, it has become clear that some of the modifications designed to improve titre, actually facilitated butanol production in the absence of an exogenous pathway. For instance, deletion of *ADH1*, a gene encoding the major alcohol dehydrogenase for ethanol production activates an endogenous butanol production pathway (Si et al. 2014; Swidah et al. 2015). It has been proposed that this pathway stems from threonine catabolism in mitochondria and borrows enzymes from the leucine biosynthetic pathway (Si et al. 2014); hence, it has been termed the threonine-based ketoacid (TBK) pathway (Shi et al. 2016) (Fig. 1). Indeed, overexpression of appropriate pathway genes and elimination of competing pathways further improved the butanol titre (Si et al. 2014; Shi et al. 2016). Intriguingly, the existence of an endogenous butanol pathway connected with hydrophobic amino acid metabolism was suggested as early as the 1960s (Ingraham et al. 1961). The combination of endogenous and exogenous pathways within butanol producer strains likely represents a fundamental strategy for the further enhancement of butanol titre (Shi et al. 2016; Swidah et al. 2015)*.*

Another pathway that has been studied in yeast with a view to butanol production is a glycine-butanol (GB) pathway. Here, butanol is produced when glycine is used as a substrate in the fermentation media (Branduardi et al. 2013). Once again, a combination of exogenous and endogenous enzymes improves butanol production, as introduction of *goxB* from *B. subtilis* enhances the conversion of glycine into glyoxylate, which is subsequently converted to butanol in a series of reactions (Fig. 1).

In previous work, we have engineered a strain of *S. cerevisiae* for n-butanol production. We showed that the butanol is synthesised in parallel from an endogenous butanol synthetic pathway activated by *ADH1* deletion and a heterologous ABE synthetic pathway. Combining these pathways with a strategy to channel the carbon flux toward acetyl-CoA production by overexpressing *ALD6 ACS2* further enhanced titre (Swidah et al. 2015). In this current study, we have characterised the relative contribution of the endogenous and exogenous pathways involved in n-butanol production in these yeast strains. We present various experiments suggesting that the exogenous pathway is responsible for much of the butanol production in our strains. Endogenous butanol production that is activated by *ADH1* deletion does not rely upon leucine biosynthetic enzymes and so the threonine-based ketoacid (TBK) pathway is unlikely to play a role. In attempts to characterise potential routes for endogenous butanol production, different substrates were added to the fermentation media. Butanol titres were increased when glycine was used as a supplement, and exceeded 2400 mg/L when α-ketovalerate was supplied. Therefore, yeast harbour the enzymatic machinery required for butanol production, and the characterisation and subsequent optimisation of this machinery provides intriguing avenues for further research into yeast as a cellular biofuel factory.

## Methods

### Yeast growth and engineering

All the engineered yeast used in this work were derived from *S. cerevisiae* W303-1A (ATCC: MYA-2181). Yeast were grown at 30 °C on standard yeast extract/peptone/dextrose (2% *w*/*v*) media (YPD) (Guthrie and Fink 1991). In the supplementation experiments, α-ketovalerate (Sigma-Aldrich) and glycine were added to YPD at 0.2%. For α-ketovalerate, the media was buffered at pH 5.0 using a citrate/phosphate buffer. The *MLS1* and *CIT2* genes were deleted using standard yeast PCR-based gene disruption methods and the *loxp-natNT2-loxp* marker from the pZC2 vector (Carter and Delneri 2010). Subsequent removal of the *natNT2* marker was achieved using a cre recombinase strategy, which allowed construction of the double *mls1Δcit2Δ* mutants. The *LEU2* prototroph was generated by transformation with an *Ssp*I 1.5-kb fragment *LEU2* fragment isolated from the pRS vector. The *LEU1* and *LEU4* genes were deleted using the *loxp-natNT2-loxp* cassette using standard yeast PCR-based gene disruption methods. Genotype alterations were verified by PCR analysis on genomic DNA and assessment of known phenotypic consequences.

### Measurements of butanol and ethanol

Yeast were grown as semi-anaerobic batch cultures from a starting OD_600_ of 0.1 in liquid YPD media using 50-ml vials over a 21-day period. Media samples were taken, passed through a 0.22-μ filter into gas chromatography (GC) vials and analysed by GC-FID using an Agilent 6850A GC system with an Agilent 4513A automatic injector, sampler and controller. A DB-WAX capillary column (30 m × 0.25 mm, 0.25 μM) was used for separation. Samples were collected every 2 days and quantified relative to standards of ethanol and butanol. Where specific values are shown, levels on days 11 or 13 were used.

### Western blot analysis of flag-tagged proteins

Yeast cultures (5 ml) were grown to an OD_600_ of 0.7 in YPD, pelleted; then protein samples were prepared and processed for electrophoresis and immunoblot analysis as described previously (Ashe et al. 2000) using an Anti-Flag antibody (Sigma-Aldrich).

## Results

### The ABE pathway explains a major part of the butanol production in engineered *S. cerevisiae*

Previously, we have described an engineered version of W303-1A (termed *A6A2adh1Δ + 5g*) that produces substantial levels of butanol. In this strain, five butanol production enzymes (four from *Clostridia—*Hbd, Crt, Bcd and Adhe2*—*plus one from *S. cerevisiae*—Erg10p) and the enzymes for cytosolic acetyl-CoA synthesis (Ald6p and Acs2p) are overexpressed, while the major alcohol dehydrogenase *ADH1* is deleted (Fig. 1). Intriguingly, the *ADH1* deletion by itself, without other strain alterations, leads to low level butanol production (Swidah et al. 2015). This finding is consistent with what has been shown by other investigators and is suggestive that endogenous pathways for butanol production exist in yeast (Si et al. 2014)*.* The deletion of *ADH1* also causes a range of physiological alterations including significant reductions in ethanol production, the accumulation of acetaldehyde and acetate and a decrease in cellular growth rate (de Smidt et al. 2012; Swidah et al. 2015). The presence of the butanol production enzymes in an *adh1Δ* strain led to minor restorative effects for some of these phenotypes: for instance, the growth rate was slightly improved (Swidah et al. 2015). This means it is formally possible that the enzymes of the heterologous butanol production pathway are not responsible for butanol production per se. Instead, the endogenous pathway that produces low levels of butanol in the *adh1* deletion strain is somehow enhanced by the presence of these enzymes: perhaps simply by improving the growth of the strain. Therefore, a key question is whether the heterologous ABE pathway that has been introduced into *S. cerevisiae* is active and how much it contributes to butanol levels relative to potential endogenous butanol production pathways. To address this, a genetic strategy was taken where the effect of having four of the five butanol production pathway genes was assessed in strains carrying the *ADH1* deletion and overexpressing *ALD6* and *ACS2*.

**Fig. 2:**
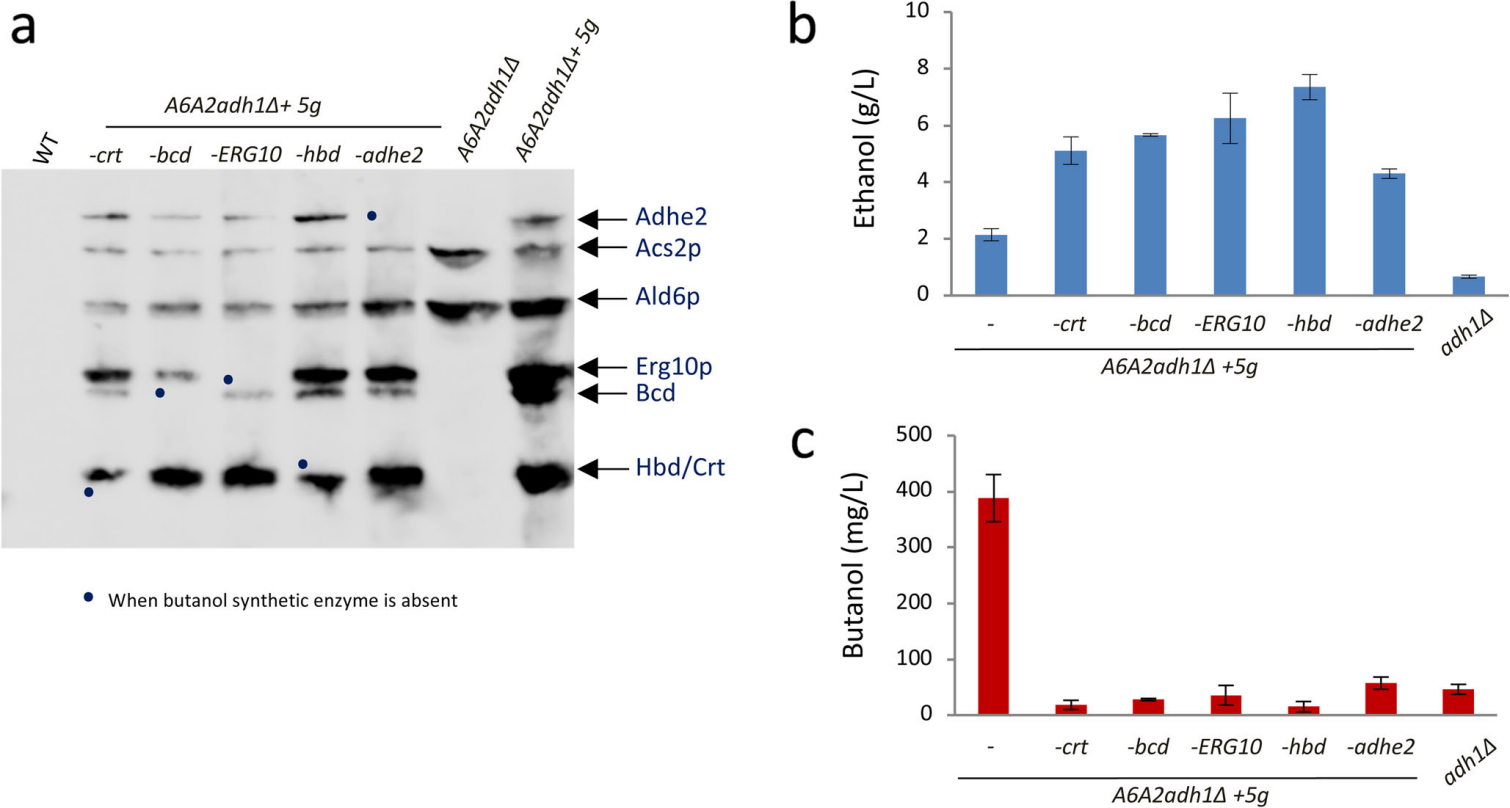
The exogenous ABE pathway is a major factor defining butanol levels in the butanol production strain. **a** Western blot using an anti-Flag antibody to detect protein expression from protein extracts prepared from the indicated strains. The butanol production strain—*A6A2adh1∆ + 5 g* (carrying the five enzymes of the ABE pathway, overexpressing Ald6/Acs2p and deleted for *ADH1*), the wild type parent (WT) and the *A6A2adh1∆* strain (overexpressing Ald6/Acs2p and deleted for *ADH1*) are used as controls. They are compared to five strains derived from the production strain, but each lacking one of the proteins from the ABE pathway (-*crt*, -*bcd*, -*ERG10*, -*hbd1* and -*adhe2*). Proteins derived from the five integrated cassettes of the ABE pathway and the integrated cassette overexpressing Acs2p and Ald6p are labelled on the right. Bullets represent the position where the expected protein is absent from the blot. **b**, **c** Graphs depicting the level of ethanol or butanol from the constructed strains on day 14 using semi-anaerobic fermentation conditions. Error bars are ± SD from 5 biological repeats

Evaluation of the expression levels of the various enzymes in these strains confirmed that all of butanol synthetic enzymes as well as Ald6p and Acs2p enzymes were highly expressed in the parent strain (*A6A2adh1Δ+5g*) (Fig. 2a). In contrast, expression of one of the enzymes (Crt, Bcd, Erg10p, Hbd or Adhe2) was deficient in the appropriate deletion strain (Fig. 2a). As described previously for the *A6A2adh1Δ+5g* production strain, ~300–400 mg/L butanol was produced over the course of a fermentation, whereas the *adh1Δ* strain produced < 100 mg/L butanol. In all of the strains carrying four of the five butanol production pathway enzymes, the levels of butanol produced were similar or less than that produced by the *adh1Δ* strain (Fig. 2c). Interestingly, across the fermentations for the constructed mutants, the levels of ethanol are higher compared to the parental *A6A2adh1Δ+5g* strain or the *adh1Δ* strain (Fig. 2b). It is possible that in the absence of the butanol production pathway, any acetate, acetaldehyde or acetyl-CoA that accumulate due to the deletion of *ADH1* and overexpression of *ALD6*/*ACS2* gets converted to ethanol over the course of the fermentation, whereas the presence of an active butanol production pathway provides a driving force minimising back flux to ethanol.

Overall, these data suggest that in the parent strain producing high levels of butanol, the heterologous butanol production pathway is active, all five components of the pathway are required for activity and the pathway explains a large proportion of the butanol synthesised by the strain. Residual butanol production is apparent and this presumably stems from an endogenous butanol production pathway.

### Mutations to the glyoxylate shunt pathway inhibit the heterologous butanol production pathway without affecting the endogenous pathway

**Fig. 3:**
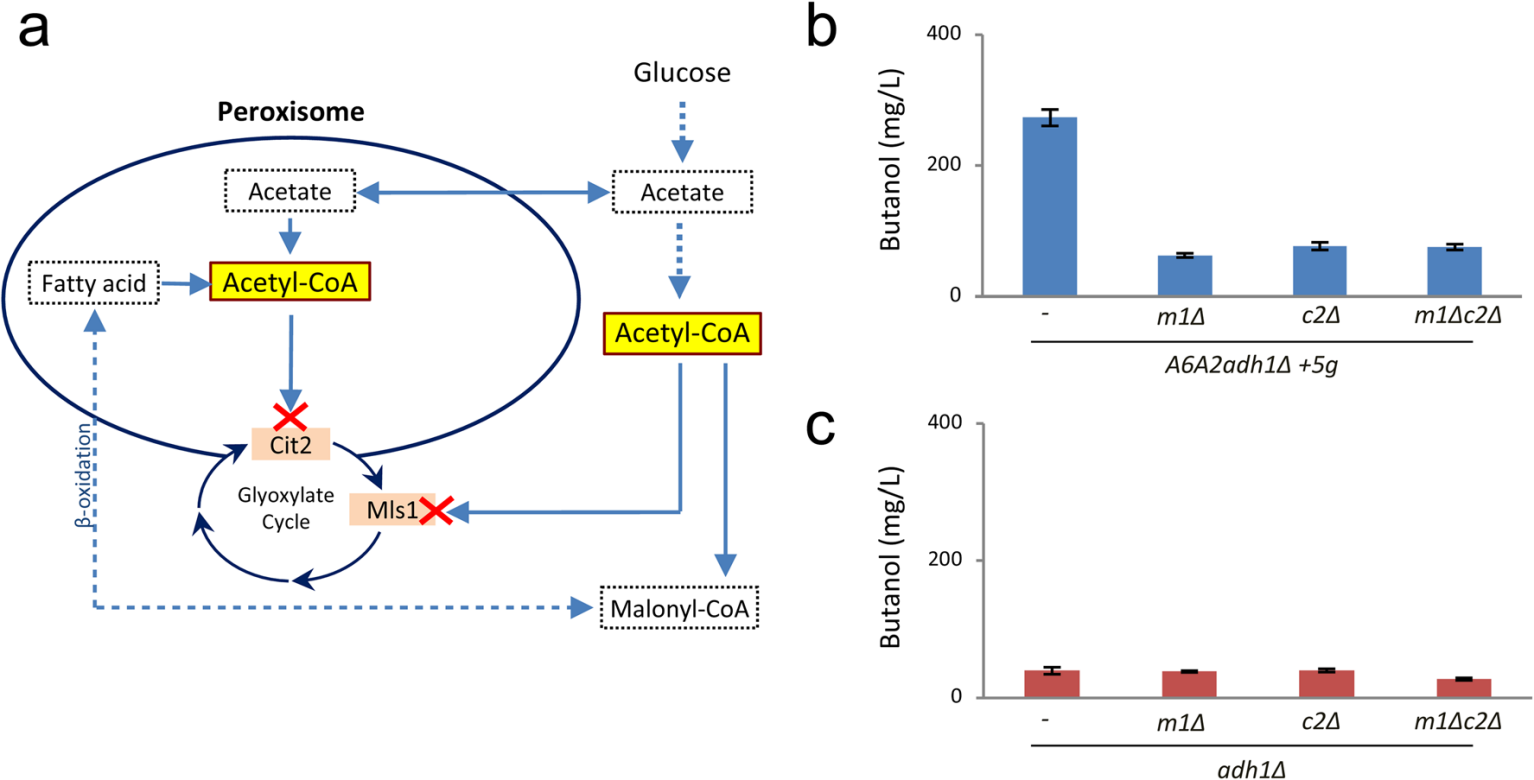
Acetyl-CoA consuming reactions from the Glyoxylate cycle impact on the exogenous but not the endogenous butanol pathway. **a** Schematic diagram of the glyoxylate cycle in yeast focussing on acetyl-CoA and reactions that impact upon its levels in the cytosol and peroxisome (Hiltunen et al. 2003). The reactions targeted in the deleted strategy to increase the pool of acetyl-CoA are marked with a red cross **b, c** Bar charts show butanol levels for single and double deletions of *MLS1* and *CIT2* in either the butanol production strain (*A6A2adh1∆ + 5g*) or the *adh1∆* strain from semi-anaerobic fermentations

During the course of studies aimed at optimising butanol synthesis from the butanol production strain *A6A2adh1Δ+5g*, further evidence was obtained supporting the existence of both heterologous and endogenous butanol production pathways. Initially, we reasoned that increasing cytosolic acetyl-CoA might lead to higher butanol levels from our production strain. Previous studies have shown deleting the glyoxylate shunt genes, *MLS1* and *CIT2*, encoding acetyl-CoA depleting components (Fig. 3a), leads to greater butanol production in the context of strains overexpressing a number of other yeast and Clostridial genes (Krivoruchko et al. 2013). Therefore, *MLS1* and *CIT2* were deleted singly or in combination in both our butanol production strain and the *adh1Δ* strain. However, in the context of our butanol production strain *A6A2adh1Δ+5g*, no improvement in butanol titre was evident in the deletions. In fact, the deletions caused a large reduction in butanol titre (Fig. 3b). Notably though, in the *adh1Δ* strain where the source of butanol is an endogenous pathway, the deletions in *CIS2* or *MLS1* had no impact on butanol levels (Fig. 3c). Equally, the deletions reduce butanol production in the *A6A2adh1Δ+5g* strain to the level observed in the *adh1Δ* strain, suggesting that the endogenous pathway is still active in this strain. Therefore, these mutant data are suggestive that there are two distinct butanol pathways, the heterologous butanol production pathway that is sensitive to alterations to the glyoxylate shunt pathway, and the endogenous pathway that is insensitive to these gene deletions.

The question as to why deletion of glyoxylate shunt components in our *A6A2adh1Δ+5g* producer strain reduces butanol production, specifically from the heterologous pathway, while in other strain contexts it activates butanol production (Krivoruchko et al. 2013), likely reflects the distinct context in the relative strains. In our strain, flux toward ethanol has been inhibited by deletion of *ADH1*, which likely leads to the accumulation of toxic intermediates such as acetaldehyde and acetate. Deletion of *MLS1* and *CIT2* might further exacerbate this effect by stemming depletion of cytosolic acetyl-CoA, which may reduce both growth and butanol production. Indeed, the accumulation of biomass was somewhat delayed in the *MLS1*/*CIT2* deletion strains relative to the parent (data not shown). In contrast, in studies where deletion of *MLS1* and *CIT2* improved butanol titre (Krivoruchko et al. 2013), *ADH2* was overexpressed to drive production of acetaldehyde from ethanol. It is unlikely that such overexpression in the context of an active *ADH1* gene (which catalyses the reverse reaction) would lead to the same accumulation of acetaldehyde and acetate. Therefore, in this strain, the *MLS1* and *CIT2* deletions may have the desired effect of leading to accumulation of cytosolic acetyl-CoA: the substrate for the butanol pathway.

### A previously characterised endogenous butanol pathway does not explain the butanol accumulation

*S. cerevisiae* can accumulate up to 120 mg/L n-butanol after deletion of the *ADH1* gene (Si et al. 2014; Swidah et al. 2015). Previous studies have suggested that an endogenous threonine-based ketoacid (TBK) pathway is involved in this butanol production, where threonine catabolism to 2-ethylmalate in the mitochondria precedes cytosolic conversion to n-butanol through the action of various components of the leucine biosynthetic pathway (Si et al. 2014; Shi et al. 2016). The leucine biosynthetic genes Leu1p, Leu2p, Leu4p and Leu9p form part of this native pathway (Fig. 1).

**Fig. 4:**
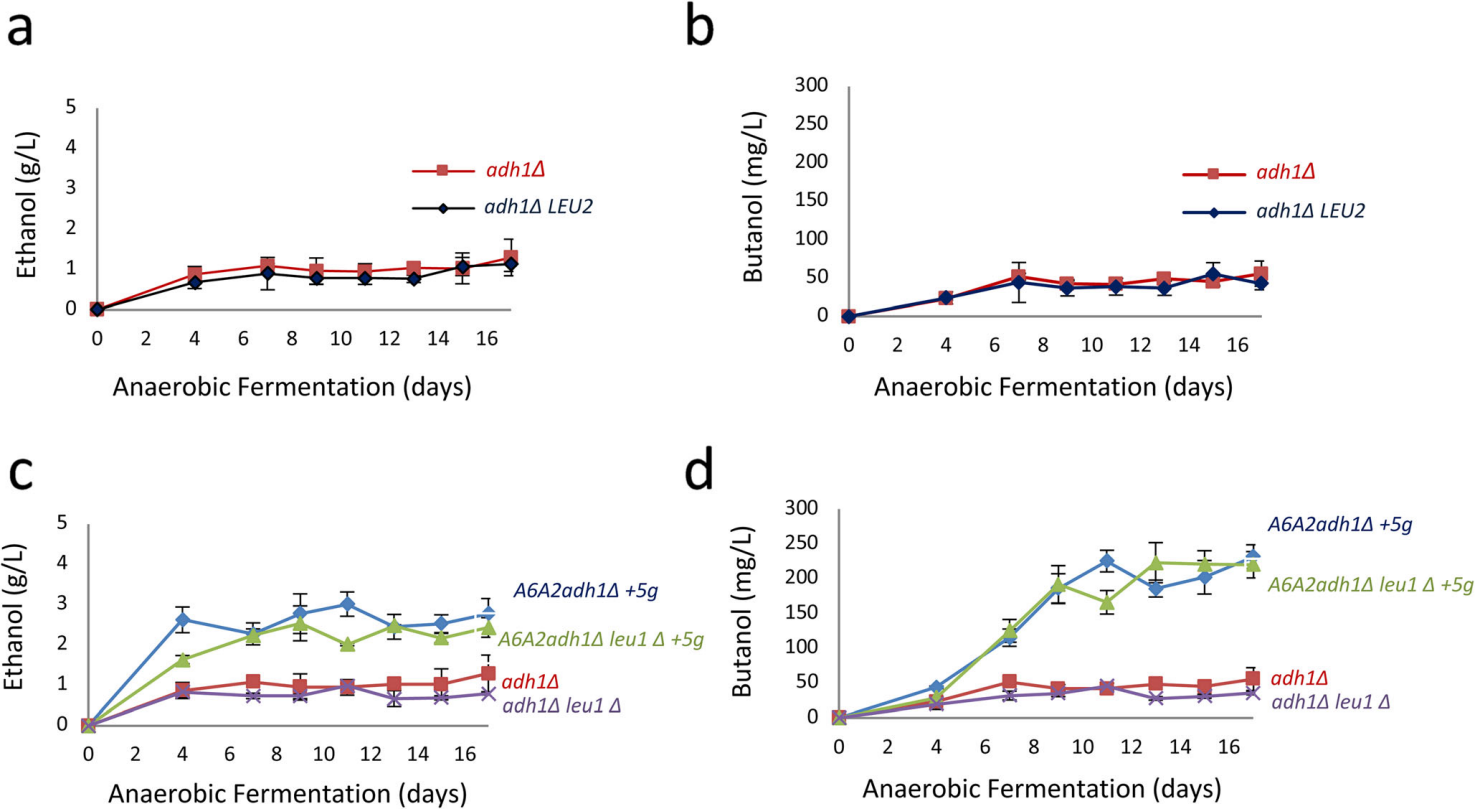
Elements of the Leu pathway are not involved in the endogenous pathway of butanol production. A. & C. Graphs depicting the level of ethanol and butanol after rescuing the *LEU2* gene in *adh1∆* strain over 18 day semi-anaerobic fermentations. D. & E. Graphs depicting the level of ethanol and butanol after deleting *LEU1* in *adh1∆* strain and butanol production strain (A6A2*adh1∆ + 5 g*) over a 18 days using semi-anaerobic fermentation conditions. Error bars are ±SD from 5 biological repeats

Previously, we found that an *adh1Δ* mutant strain produces n-butanol in the absence of other alterations, even though the strain is a leucine auxotroph carrying the *leu2-3,112* mutant allele (Swidah et al. 2015). Leu2p is a β-isopropylmalate dehydrogenase that is also capable of converting 3-ethylmalate to α-ketovalerate as part of a threonine-based ketoacid pathway (Shen and Liao 2008; Si et al. 2014; Branduardi et al. 2013; Andreadis et al. 1984). In order to assess whether this reaction was important for the endogenous pathway in our strains and if so, to improve the efficiency of butanol production, we undertook a strategy to rescue the *LEU2* gene in the *adh1Δ* strain background. In order to generate this *LEU2* prototrophic strain, a *LEU2* rescue fragment was isolated, transformed into the *adh1Δ* strain, screened on selective media and validated for integration at the endogenous locus. Intriguingly, rescuing *LEU2* in the *adh1Δ* strain had no impact on n-butanol or ethanol production (Fig. 4a, b). However, it seemed plausible that in our *adh1Δ* strain another enzyme could be catalysing this reaction in the absence of Leu2p. Interestingly, previous authors have also speculated that residual activity in a *LEU2* deletion strain could be due to other enzymes (Branduardi et al. 2013).

A prediction of this would be that deletion of other leucine biosynthetic genes in the threonine-based ketoacid pathway, such as *LEU1*, would block butanol production from the endogenous pathway. This would be expected to decrease the n-butanol titre considerably for the *adh1Δ* strain and only slightly for *A6A2adh1Δ+5g* production strain. Leu1p is a cytosolic FeS dehydratase that can catalyse the conversion of 2-ethylmalate into 3-ethylmalate, as well as the second step in the leucine biosynthetic pathway (Skala et al. 1991). Although, the mitochondrial aconitase and homoaconitase enzymes of the TCA cycle and lysine biosynthetic pathway, respectively, are from the same family as Leu1p they have very distinct substrate characteristics and subcellular localization (Lill et al. 1999). Therefore, the *LEU1* gene was deleted in the *adh1Δ*, *A6A2adh1Δ+5g* and parent strains, and the resulting strains were confirmed as leucine auxotrophs. Surprisingly, deletion of *LEU1* did not decrease ethanol or n-butanol levels for the *leu1Δadh1Δ* strain compared to the parent *adh1Δ* strain (Fig. 4c). Equally, *LEU1* deletion had little effect on titre from the *A6A2adh1Δ+5g* strain (Fig. 4d). Similar observations were made when the *LEU4* gene was deleted (data not shown). Leu4p is the major isozyme responsible for the first step in the leucine biosynthesis pathway and can convert α-ketobutyrate into 2-ethylmalate in the mitochondria (Drain and Schimmel 1988; Baichwal et al. 1983; Beltzer et al. 1988). Our results suggest that specific enzymes of the leucine biosynthetic pathway are not required for butanol production from an endogenous pathway that is activated after deletion of *ADH1*.

### α-Ketovalerate is efficiently converted to n-butanol

**Fig. 5:**
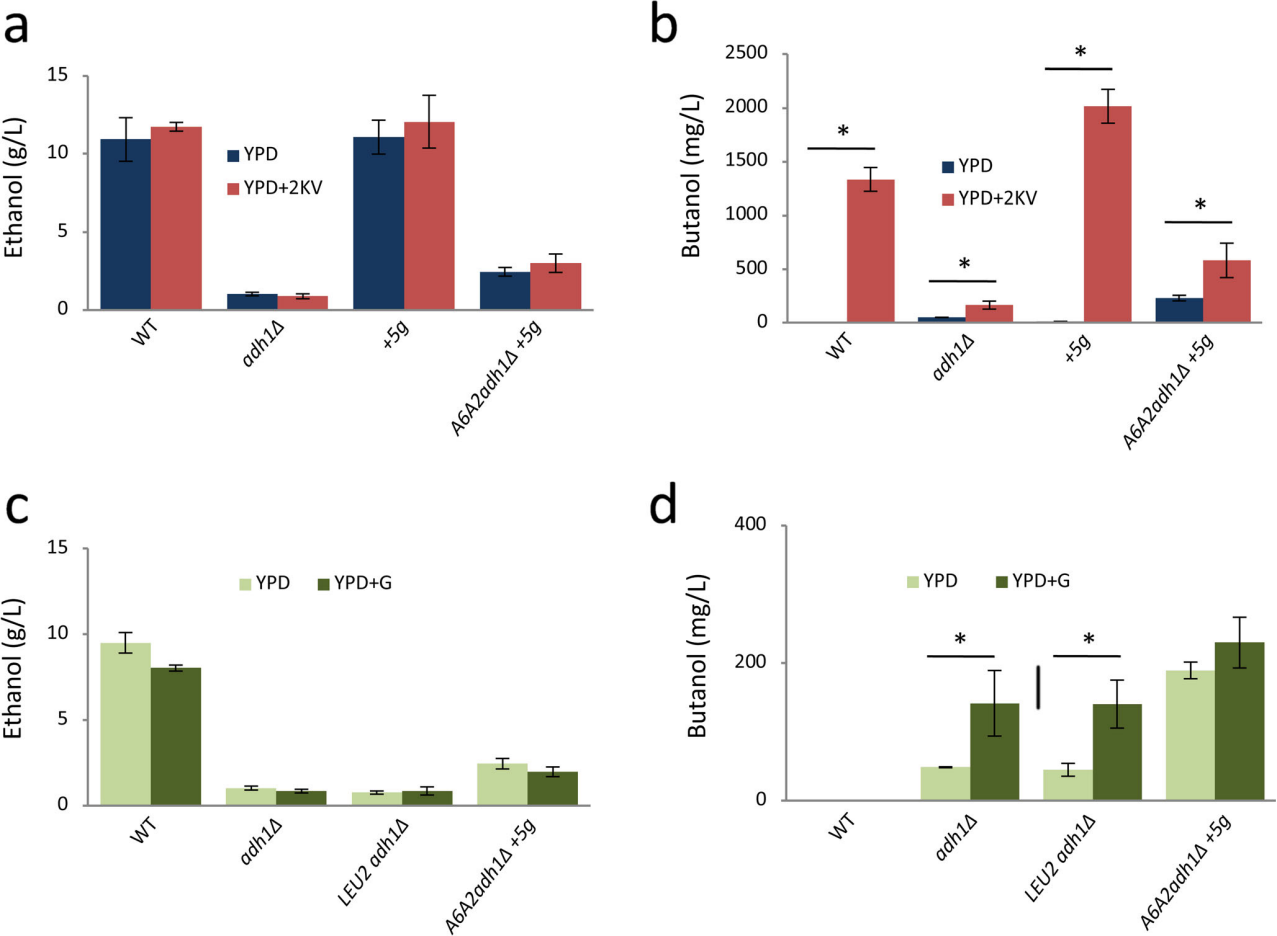
Using α-ketovalerate or glycine in the growth media significantly improves butanol production*.* Bar charts depict the results of fermentation experiments in YPD medium with and without 0.2% α-ketovalerate (**a, b**) or 0.2% glycine (**c, d**) as indicated. The level of ethanol (A and C) or butanol (**b, d**) was quantified on day 13 of a standard semi-anaerobic fermentation. Error bars are ± SD from 5 biological repeats. For selected comparisons * represents a difference where *p* < 0.05

Two butanol production pathways have been described where much or the vast majority of the pathway relies upon endogenous enzymes, the TBK pathway described above and the glycine-butanol pathway (Si et al. 2014; Shi et al. 2016; Branduardi et al. 2013). A common element to both of these pathways is the conversion of α-ketovalerate to n-butanol. This conversion would require an α-keto acid decarboxylase (KDC) to generate butyraldehyde and an alcohol dehydrogenase to give n-butanol (Romagnoli et al. 2012). In order to assess whether in our strains enzymes that would enable such a conversion are active, we added α-ketovalerate to the fermentation media. While all of the strains tested produced expected levels of ethanol in rich media (YPD) and rich media with α-ketovalerate (Fig. 5a, b), the addition of α-ketovalerate not only improves butanol production for the strain lacking *ADH1* (up to 140 mg/L for *adh1Δ* and 600 mg/L for *A6A2adh1Δ+5g* production strain), but also leads to a marked improvement for the strains harbouring functional *ADH1*: the WT produces (1.4 g/L), whereas the strain bearing 5 butanol synthetic genes produces (2.4 g/L). It is entirely possible that the variation in butanol levels for these strains is relevant to the presence of the ABE pathway in the butanol production strain. For instance, strains carrying the ABE pathway contain the Adhe2 enzyme, which could participate in boosting the butanol titre compared to the parent strain.

These results not only highlight that the enzymes responsible for converting α-ketovalerate to n-butanol are present in yeast, but that there is potential for increasing butanol titre significantly by targeting endogenous pathways of butanol production, particularly in combination with exogenous pathways.

### Glycine is a likely source of endogenous n-butanol

Previous studies have shown that *S. cerevisiae* strains are able to accumulate n-butanol from glycine as a nitrogen source (Branduardi et al. 2013). To enable slightly higher levels of production, a *Bacillus subtilis* glycine oxidase (*goxB*) gene was introduced into yeast to enhance the conversion of glycine to glyoxylate, which could then be converted to n-butanol via α-ketovalerate (Branduardi et al. 2013) (Fig. 1). In yeast, generally, glycine is synthesised from serine, which stems from glycolytic intermediates (Hahn 1993). Given the potential for a glycine-butanol (GB) pathway, we decided to explore the impact of using glycine as substrate on n-butanol production in our system. Interestingly, for the mutants bearing only the native butanol production pathway (*adh1Δ* and *adh1ΔLEU2*), we found that the addition of glycine improves n-butanol production significantly (40 mg/L to 120 mg/L). The levels of n-butanol were also slightly increased for the *A6A2adh1Δ+5g* production strain bearing both exogenous and endogenous pathways, although this increase was not statistically significant (Fig. 5c, d).

## Discussion

Previously, we have engineered the *S. cerevisiae* W303-1A strain with a view to the production of the key commodity chemical and biofuel, n-butanol (Swidah et al. 2015)*.* Here, we show that two parallel pathways contribute to this production: an endogenous pathway activated after deletion of the major alcohol dehydrogenase, *ADH1*, and an exogenous ABE-based pathway, which together have the capacity to produce 300–380 mg/L butanol.

Using genetic analysis, we show that the heterologous ABE pathway is producing the majority of the butanol in the production strain. Firstly and most significantly, deletion of any single gene from the ABE pathway is entirely sufficient to reduce butanol levels to the base level produced when *ADH1* alone is deleted. Second mutations in the glyoxylate pathway impact upon production via the ABE pathway enzymes without effecting production from an *ADH1* deletion strain. These data combined strongly suggest that two parallel butanol production pathways are present and active within our strains. This agrees with other recent studies highlighting the value in terms of butanol titre in combining different strategic approaches for butanol production within a single strain (Shi et al. 2016).

The presence of an endogenous pathway within our *adh1Δ* strain, however, presented a conundrum based upon other studies with *adh1Δ* mutant strains (Si et al. 2014). As seemingly, the most likely route for butanol production is via the threonine-based ketoacid pathway involving a number of leucine biosynthetic enzymes including Leu2p (Si et al. 2014; Ingraham et al. 1961), whereas our *adh1Δ* strain carries a non-reverting auxotrophic allele of the *LEU2* gene (Hinnen et al. 1978). We considered that perhaps another activity might be compensating for the Leu2p enzyme in our yeast strains. With this in mind, we replaced the auxotrophic *LEU2* allele with the wild type expecting an improvement in butanol levels. However, no such improvement was evident. Equally, deletion of either the *LEU1* or *LEU4* genes higher in the pathway than *LEU2* did not reduce butanol production in any way. On this basis, we conclude that the TBK pathway is not involved in butanol production in our *adh1Δ* strain.

Another study has described butanol production from glycine (Branduardi et al. 2013). In this study, the hypothesis was put forward that glycine could be converted to butanol via conversion to glyoxylate, followed by glyoxylate condensation with butyryl-CoA to 3-ethylmalate and 3-ethylmalate conversion to α-ketovalerate then on to n-butanol. Interestingly, using α-ketovalerate as a precursor for butanol production in the fermentation media not only leads to improved butanol titre in all our metabolically engineered strains but also a non-engineered parental strain produced up to 2 g/L. This result shows that *S. cerevisiae* actively express enzymes capable decarboxylating and reducing α-ketovalerate to n-butanol. Previous work has suggested that a range of 2-ketoacid decarboxylase enzymes including the pyruvate decarboxylase enzymes (Pdc1p, Pdc5p or Pdc6p) would be capable of undertaking the decarboxylation reaction and a number of alcohol dehydrogenases could convert butyraldehyde to n-butanol (Romagnoli et al. 2012; Larroy et al. 2002, b). Further evidence that a pathway from glycine might be the source of n-butanol in our strain stems from the supplementation of the yeast fermentation media with glycine. Here, a 3-fold increase in the level of butanol was observed. However, the interpretation that a glycine-butanol pathway is responsible for butanol production in the *adh1Δ* strain is difficult to reconcile with a number of other observations. For instance, the Leu2p enzyme is once again thought to play a key role, catalysing the conversion of 3-ethylmalate to α-ketovalerate. Yet our strain is auxotrophic for the Leu2p enzyme, and when we complement the *LEU2* auxotrophic mutations, no effect on butanol levels was observed. However, in other studies, following deletion of the *LEU2* gene, the resulting yeast strains still harboured at least some capacity to carry out this enzymatic reaction (Branduardi et al. 2013). Therefore, it might be possible that a redundant enzyme could catalyse this reaction. Another issue with this pathway is that we have deleted the malate synthase gene, *MLS1*, and found that the *adh1Δ* strain still produces similar levels of butanol. Mls1p catalyses the condensation of butyryl-CoA with glyoxylate to give 3-ethylmalate downstream of glycine. Once again, redundancy may represent an answer, as Dal7p also catalyses the same reaction and might compensate for the lack of Mls1p. A final concern is that while several studies suggest the existence of a yeast enzyme that is capable of converting glycine into glyoxylate (Branduardi et al. 2013; Villas-Boas et al. 2005), it has not yet been identified. In previous studies, a glyoxylate oxidase from *Bacillus subtilis* was used to bolster this reaction, but without knowing the precise gene involved it is difficult to prove a role for the reaction.

In conclusion, we provide evidence that in our engineered yeast butanol production strains, two parallel pathways are important for butanol synthesis. Firstly, the introduced heterologous ABE pathway is responsible for a large part of the butanol produced. Secondly, an endogenous pathway activated by deletion of the *ADH1* gene is also responsible for butanol generation. In the interest of potentially optimising this endogenous pathway further, we sought to characterise it. Of the two previously described pathways for butanol production, our studies favour a route that stems from glycine and goes via an α-ketovalerate intermediate. Future optimisation of systems where different pathways converge on the same end product holds significant promise in the era of synthetic biotechnology.
